# Comparison of Result Times Between Urine and Whole Blood Point-of-care Pregnancy Testing

**DOI:** 10.5811/westjem.2016.5.29989

**Published:** 2016-06-22

**Authors:** Michael Gottlieb, Kristopher Wnek, Jordan Moskoff, Errick Christian, John Bailitz

**Affiliations:** John H. Stroger Hospital of Cook County, Department of Emergency Medicine, Chicago, Illinois

## Abstract

**Introduction:**

Point-of-care (POC) pregnancy testing is commonly performed in the emergency department (ED). One prior study demonstrated equivalent accuracy between urine and whole blood for one common brand of POC pregnancy testing. Our study sought to determine the difference in result times when comparing whole blood versus urine for the same brand of POC pregnancy testing.

**Methods:**

We conducted a prospective, observational study at an urban, academic, tertiary care hospital comparing the turnaround time between order and result for urine and whole blood pregnancy tests collected according to standard protocol without intervention from the investigators. After the blood was collected, the nurse would place three drops onto a Beckman Coulter ICON 25 Rapid HCG bedside pregnancy test and set a timer for 10 minutes. At the end of the 10 minutes, the result and time were recorded on an encoded data sheet and not used clinically. The same make and model analyzer was also used for urine tests in the lab located within the ED. The primary outcome was the difference in mean turnaround time between whole blood in the ED and urine testing in the adjacent lab results. Concordance between samples was assessed as a secondary outcome.

**Results:**

265 total patients were included in the study. The use of whole blood resulted in a mean time savings of 21 minutes (95% CI 16–25 minutes) when compared with urine (p<0.001). There was 99.6% concordance between results, with one false negative urine specimen with a quantitative HCG level of 81 mIU/L.

**Conclusion:**

Our results suggest that the use of whole blood in place of urine for bedside pregnancy testing may reduce the total result turnaround time without significant changes in accuracy in this single-center study.

## INTRODUCTION

Point-of-care (POC) pregnancy testing is commonly performed in the emergency department (ED). Studies have demonstrated that patient sexual history is unreliable,^1^ and many patients may need radiographic procedures or administration of potentially teratogenic medications during the course of their ED visit. In many United States EDs, pregnancy testing is performed by POC urine pregnancy testing. However, with the exception of bladder catheterization, awaiting urine specimens may result in significant delays if the patient is not yet able to provide urine, or may be impossible if the patient is anuric due to illness or injury. Fromm et al previously demonstrated in 633 patients that urine and whole blood have similar test characteristics when used in one common brand of POC pregnancy testing, with a higher sensitivity and a lower human chorionic gonadotropin (hCG) threshold level observed in the whole blood specimen group.^2^

There has been increasing pressure to increase efficiency and throughput in the modern ED. Previous studies have suggested that lab turnaround times may play a significant factor in patient throughput, which can affect both ambulance diversion rates and overall departmental efficiency.^3^–^8^ Despite multiple studies assessing improvement in turnaround time when performing laboratory studies in the ED and at the bedside instead of in a separate laboratory, none have assessed the potential time savings of replacing urine pregnancy testing with whole blood. Our aim was to investigate whether the use of whole blood in place of urine for bedside POC pregnancy testing would result in a decrease in the turnaround time for results. As secondary outcomes, we assessed net decrease in turnaround time when selecting the faster result and concordance among both test results.

## METHODS

### Study Design

This was a prospective, observational study of female patients of childbearing age presenting to the ED who had both blood obtained and a pregnancy testing performed as a routine part of their care. This study was conducted at an urban, academic, tertiary care hospital with an annual census of 110,000 patients per year.

### Study Setting and Population

All female patients aged 18 to 55 years who presented to the ED and had both blood drawn and a pregnancy test ordered as a routine part of their care were eligible for inclusion. Exclusion criteria included prior hysterectomy, known or obvious pregnancy, hemodynamic instability, blood obtained prior to the placement of any orders, and presentation when one of the trained nurses was not available.

The study complied with the recommendations of the Strengthening the Reporting of Observational Studies in Epidemiology (STROBE) statement.^9^ The study was approved by the local institutional review board with waiver of informed consent. There was no manufacturer support for this study and none of the study investigators have conflicts of interest to declare.

### Study Protocol

All blood was obtained per standard nursing protocol without intervention by the study team. Patients were only enrolled if they were having blood drawn for non-pregnancy purposes. Once blood was obtained, three drops from a syringe were placed onto a Beckman Coulter ICON 25 Rapid hCG bedside pregnancy test and a timer was set for 10 minutes. Blood was placed directly into the Beckman Coulter ICON 25 Rapid hCG bedside pregnancy test without any special handling or centrifugation. The Beckman Coulter ICON 25 Rapid hCG POC pregnancy tests were already stocked in this ED and required no special machinery and limited provider training. The decision to wait 10 minutes was based upon the prior accuracy study protocol from Fromm et.^2^ After 10 minutes, the time and result were recorded in a study binder. The blood results were not used clinically in any manner. Urine was also collected and brought to our ED laboratory (located next to the nursing station) for pregnancy testing as per the standard protocol at our institution. All urine POC pregnancy testing was performed using the same Beckman Coulter ICON 25 Rapid hCG bedside pregnancy test described above. Per the manufacturer lab manual, the threshold for positivity for this test is 25 mIU/mL.

### Outcome Measures

The primary outcome of this study was the difference in turnaround time between POC whole blood and POC urine pregnancy test results. We calculated the whole blood turnaround time as the time difference between when the first blood order was placed (obtained via electronic timestamp of order placement) and the result time (as noted by the study nurses in the binder). The urine turnaround time was calculated as the difference between when the urine pregnancy test order was placed and when the result was made available to the physician in the computer (both obtained via electronic timestamps). Our electronic medical records system allows the user to identify when laboratory results are specifically made available for the provider to view them, thereby allowing for a more accurate measurement of turnaround time. Secondary outcomes included an assessment of the concordance between urine and whole blood POC pregnancy test results and a comparison of turnaround times when selecting the faster alternative across all samples.

### Data Analysis

We calculated a sample size of 225 subjects based upon a 90% power with a two-tailed alpha=0.05 to detect a difference of 15 minutes in the turnaround time, which was estimated to be the lowest clinically significant difference and was confirmed with pilot testing. Mean values were calculated with 95% confidence intervals and compared using a paired t-test.

## RESULTS

**Figure 1:**
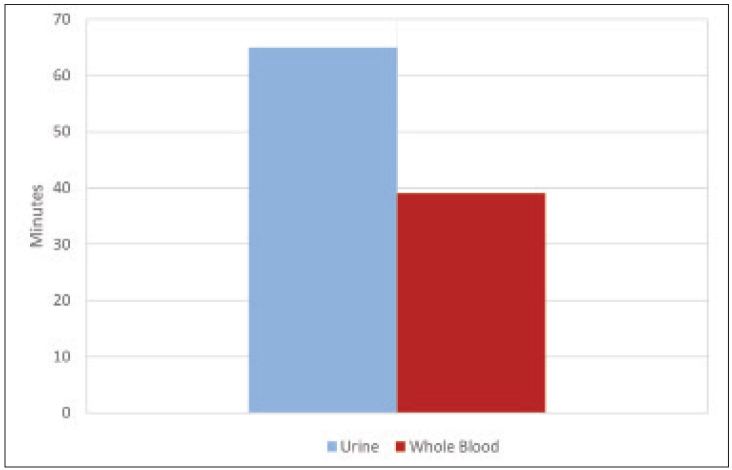
Difference in result turnaround times between whole blood and urine pregnancy tests.

**Figure 2:**
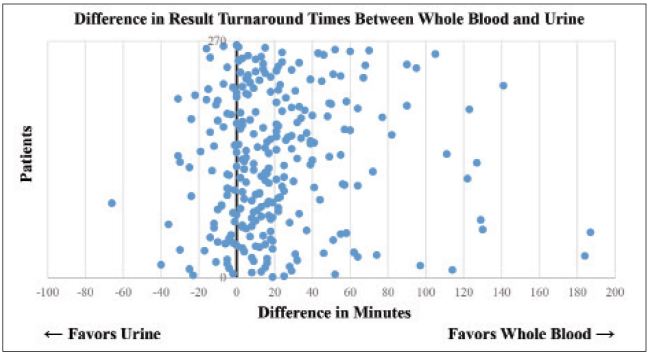
Scatter plot demonstrating the differences in the turnaround time between whole blood and urine pregnancy tests.

We obtained 265 total samples with 87 (32.8%) positive urine pregnancy tests and 178 (67.2%) negative urine pregnancy tests; 173 (65.3%) were obtained during the morning shift (07:00–15:00), 80 (30.2%) were obtained during the afternoon shift (15:00–23:00), and the remaining 12 (4.5%) were obtained during the overnight shift (23:00–07:00). The use of whole blood resulted in a mean time savings of 21 minutes (95% CI 16–25 minutes) when compared with urine (Figure 1 and 2) (p<0.001). Urine turnaround time was faster in 204 patients, with an average time savings of 31 minutes, while blood turnaround time was faster in 61 patients, with an average time savings of 12 minutes. When assessed according to shift, no significant difference was noted.

When selecting the faster alternative across all samples, the mean time savings for the whole blood group increased to 26 minutes (95% CI 23–30 minutes). Of interest, the maximum time differences ranged from 40 minutes in favor of the urine pregnancy test to 187 minutes in favor of the whole blood pregnancy test.

Concordance between samples was 99.6%. The single discordant value was a woman following up after a completed abortion who mistakenly had a urine pregnancy test ordered. Both tests were obtained and she had a positive whole blood pregnancy test and a negative urine pregnancy test. Quantitative serum hCG testing was also obtained on the patient and was determined to be 81 mIU/mL, thus demonstrating that the urine test was a false negative.

## DISCUSSION

In United States EDs, POC pregnancy testing is common and it is important to know a patient’s pregnancy status prior to obtaining certain radiographic studies or administering a number of medications. However, this is often contingent upon the patient providing a urine sample, which may result in prolonged result times and ED stays. Given increasing concerns about ED crowding, there have been multiple studies assessing mechanisms to improve throughput.^3^–^8^ Our study provides the first assessment of replacing urine with whole blood in POC pregnancy testing to compare result turnaround times and is only the second study comparing these modalities.

In our study, we found that replacing urine with whole blood for POC pregnancy testing resulted in a mean decrease of 21 minutes in result turnaround times. More interestingly, the range of maximum turnaround times for results ranged from 40 minutes in favor of the urine pregnancy test to 187 minutes in favor of the blood pregnancy test and preferentially selecting the faster alternative across all samples resulted in a mean time savings of 26 minutes. After thorough discussion with the nurses involved in the study, the longer delays in blood most commonly involved multiple orders being placed at the same time on different patients, while longer delays in urine were predominately secondary to delays in patients providing the urine specimen. This suggests that although replacing urine with whole blood resulted in a decreased turnaround time, the largest benefit may be in providing the option to run whichever is available first.

With regards to concordance, the data demonstrated a 99.6% concordance rate between whole blood and urine. The single discordant value was a positive whole blood result and negative urine result, which was subsequently demonstrated to be a false negative urine result. It is important to note that concordance was a secondary outcome and that quantitative hCG testing was not sent out on most patients. However, Fromm et al previously assessed test accuracy as a primary outcome in a large group of patients, demonstrating similar test characteristics with a slightly improved sensitivity and decreased hCG threshold noted in the whole blood sample.^2^ One potential reason for the improved sensitivity in the whole blood specimen is the decreased dilution of whole blood samples compared with urine when patients are given large quantities of water prior to obtaining the urine sample.

It is important to note that the use of whole blood for POC pregnancy testing is not FDA approved. Our study does support one prior study^2^ that demonstrated similar accuracy. Moreover, our study is the first to support the potential for significant time savings. It is our hope that this will incite further research and encourage this or other companies to apply for FDA approval of the use of whole blood for pregnancy testing.

## LIMITATIONS

A number of potential limitations to this study must be considered. This was a prospective, observational trial and, therefore, it is not known whether these results would be re-demonstrated in a randomized controlled trial. Despite initial data suggesting equivalent test characteristics to whole blood,^2^ this product is not yet FDA approved for whole blood and consenting each patient for use of a non-FDA-approved product would have been likely to alter the true result times. Therefore, it was not feasible to perform a randomized controlled trial at this time. Additionally, this was performed at a single, large, county hospital and may not be applicable to other ED settings. Further studies will be necessary to validate these findings at other sites.

This was also a convenience sample obtained only when trained nurses were available, so it is possible that there may be a selection bias present. Although it would have been preferable to perform this study with the entire nursing staff, our resources would only allow us to perform this using the nurses in the fast track and intermediate acuity areas of the ED. However, the involved nurses were blinded to the study outcome and instructed not to alter any of their collection techniques. Additionally, there were a disproportionate number of morning and evening shifts compared with overnight shifts, so this may not apply to patients presenting overnight. However, nurses were selected who worked all three shifts and were instructed to include all patients on their shifts regardless of the time. It is also known that additional factors, such as crowding and staffing, may affect lab turnaround times. However, since both tests were performed on the same patient in close proximity, these are unlikely to have significantly influenced the difference in turnaround times.

Additionally, because the product was not FDA approved for whole blood, we were unable to perform the whole blood testing in our ED laboratory. However, the whole blood samples were performed at the nursing station, which is in close proximity to where the ED laboratory is located and is unlikely to have significantly influenced time. Moreover, since the whole blood testing was performed by nurses working clinically, as opposed to a dedicated laboratory technician, any delay would likely be in favor of the urine specimen. With regards to applicability, there is extensive evidence demonstrating that nurses can perform testing as efficiently as laboratory staff.^10^,^11^

Our study was further limited in that only one commonly used POC pregnancy test was assessed. However, this is the current test used for POC urine pregnancy testing in our ED, as well as the one studied by Fromm et al.^2^ It is possible that alternative POC pregnancy tests may demonstrate different test characteristics.

Finally, quantitative hCG testing was not sent on all patients and it is possible that there may be concordant false negative pregnancy tests; however, chart review of all cases did not demonstrate any repeat presentations for positive pregnancy testing. Moreover, the accuracy has already been assessed as a primary outcome in a prior study.^2^

Although we showed that, on balance, blood hCG testing took less time that urine, we did not study any potential downstream effects on patient length of stay or alterations in diagnostic testing.

## CONCLUSION

Our results suggest that the use of whole blood in place of urine for bedside pregnancy testing may reduce the total result turnaround time without significant changes in accuracy in this single-center study.
